# Outpatient parenteral antimicrobial therapy (OPAT) across the world: a comparative analysis—what lessons can we learn?

**DOI:** 10.1093/jacamr/dlae111

**Published:** 2024-07-19

**Authors:** Paul Reidy, Tara Breslin, Eavan Muldoon

**Affiliations:** Department of Infectious Diseases, Mater Misericordiae University Hospital, Dublin, Ireland; School of Medicine, Trinity College Dublin, Dublin, Ireland; School of Medicine, Trinity College Dublin, Dublin, Ireland; Department of Infectious Diseases, Mater Misericordiae University Hospital, Dublin, Ireland; School of Medicine, University College Dublin, Dublin, Ireland; National OPAT Programme, Health Services Executive, Dublin, Ireland

## Abstract

This paper presents a comparative analysis of Outpatient Parenteral Antimicrobial Therapy (OPAT) structures and delivery options across different countries. OPAT, a cost-effective alternative to inpatient care for patients requiring IV antimicrobial therapy, has demonstrated multiple benefits such as patient satisfaction, economic cost savings, and reduced hospital-acquired infections. Despite these advantages, there is considerable international variation in OPAT use and implementation. By examining the OPAT structures of multiple countries, we aim to identify areas of variation and explore opportunities for expansion and improvement of OPAT services.

## Introduction

Outpatient parenteral antimicrobial therapy (OPAT) refers to the administration of IV antimicrobials in an outpatient setting. OPAT can be either a transition state from an initial inpatient admission or be entirely outpatient based depending on clinical circumstances.^1^

OPAT first became a mainstream concept in the 1970s in the USA as an option for patients who required IV antimicrobials but lacked medical insurance cover. Hospital systems soon realized the advantages of OPAT in reducing costs for patients who were well enough to be at home.^1^

The benefits of an OPAT programme are multilevel. From a patient perspective it avoids the need for admission, and multiple studies have demonstrated significant patient satisfaction.^2^ Patients are often able to return to school, work or home duties, bringing societal-level economic benefits.^3^

From a hospital perspective, OPAT can create additional healthcare capacity with early discharge and admission avoidance, saving bed days and improving patient flow.^4^ OPAT dramatically reduces the cost of care associated with complex infections.^5,6^ It also reduces over-investigation, for example unnecessary daily laboratory investigations.^7^ Importantly, OPAT also reduces the risk of complications such as the acquisition of hospital-acquired infection, and exposure to MDR organisms (MDROs).

**Figure 1.**
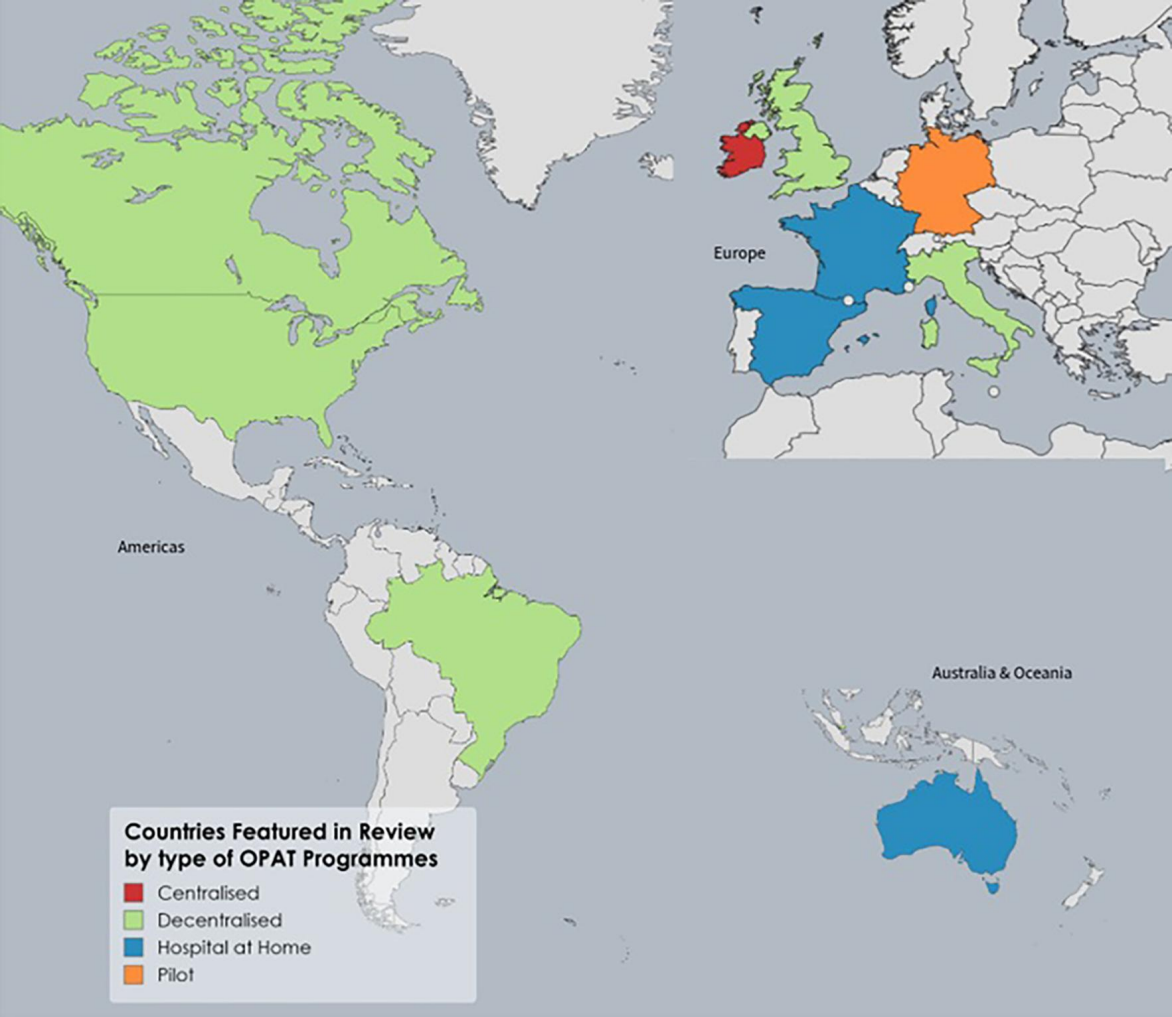
Countries featured in this review by type of OPAT programmes.

Despite the multiple benefits, there is international variation in the use and availability of OPAT. This paper seeks to outline current OPAT structures across a selection of countries with published service data and explore the opportunities for the expansion and improvement of OPAT. The list of countries is not exhaustive, but countries were selected to illustrate the differing methods of OPAT delivery across the main types of healthcare systems. Included countries are highlighted in Figure 1, with Table 1 highlighting selected OPAT characteristics of the featured countries.

**Table 1.** Components of OPAT delivery and health system structure within discussed countries

	OPAT structure	OPAT bundle/guidelines contents					National registry	Health system financing	Beds per 1000 persons	Average bed occupancy rate (%)
		Infection specialist involvement mandated	Patient follow- up and monitoring	Published national guidelines	Patient education	Programme measures				
Ireland	Centralized national programme	Yes	Yes	Yes	Yes	Yes	Yes	Tertiary care free of charge to all users Private health insurance providers available	3	93
UK	Decentralized, >80 services	Yes	Yes	Yes	Yes	Yes	Yes	Free of charge to all UK residents Private health insurance providers available	2.5	85
USA	Decentralized No national programme	Yes	Yes	Yes	Yes	Yes	No	Individual coverage dependent on insurance coverage Reimbursement through the national health insurance programme where a healthcare provider attends the home	2.9	64.3
Canada	Decentralized. No national programme	Yes	Yes	No^a^	Yes	Yes	No	Services are funded through a decentralized healthcare insurance model Only in-hospital healthcare is publicly funded. Discharged patients’ drugs/supplies may not be covered	2.5	82.7
Australia	Via Hospital at Home services	No	Yes	No^b^	Yes	Non-specific infection control measures	No but OPAT numbers contained within Hospital at Home registry	Funded by universal healthcare system Private health insurance providers available	3.8	75.3
France	Via Hospital at Home services	Yes	Yes	No	Yes	Yes	No but OPAT numbers contained within Hospital at Home registry	Covered by statutory health insurance held by all residents	5.9	78.9
Spain	Via Hospital at Home services	No	Yes	Yes	Yes	Yes	Yes	Free of charge to all users	3	75.95
Italy	Decentralized, >100 health units/19 regions/2 autonomous regions	Yes	Yes	Yes	Yes	Yes	No	Free inpatient/primary healthcare for all Specialty visits, outpatient drugs may not be covered	3.1	78.1
Germany	Pilot programme	Yes	Yes	No^a^	Yes	Yes	No	OPAT covered by statutory health insurance held by all residents	8	79.1
Brazil	Decentralized	Yes	Yes	Yes	Yes	No	No	Free of charge to all users	2.09	60
Singapore	Decentralized	Yes	Yes	No	Yes	Yes	No but combined data across hospitals	Mixed public/private Mandatory health savings account	2.5	80

^a^Using ISDA guidelines.

^b^Regional HITH guidelines on behalf of regional health, e.g. Queensland Health.

## Methods

### Study design and data sources

This study utilized a mixed-methods design to compare OPAT across multiple countries. The study drew on a range of data sources, including a systematic review of the literature, personal communication with healthcare providers, and official health data from the Organisation for Economic Co-operation and Development (OECD).

### Sample selection and data extraction

The systematic review of the literature was conducted using the PRISMA guidelines. The search strategy included the terms ‘outpatient parenteral antimicrobial therapy’, ‘OPAT’, ‘hospital in the home’ and ‘home antibiotics’. There was a date restriction of September 2023 applied to search results. The inclusion criterion was studies that reported on the use of OPAT within an individual or multiple countries. Data were extracted from eligible studies on OPAT indications, service delivery, patient populations, treatment regimens, and outcomes where available. Non-English sources were included in this study and were translated into English using Google Translate.

OPAT practice guidelines from the USA (published by the Infectious Diseases Society of America), the UK (published by the British Society for Antimicrobial Chemotherapy; BSAC) and from Ireland (published by the Infectious Diseases Society of Ireland) were also used as reference documents.^8–10^

Personal communication with healthcare providers in featured countries was used to supplement the data obtained from the systematic review. Healthcare providers with experience in OPAT delivery from different countries were identified through professional networks and questioned on factors influencing OPAT delivery, including organizational structures, funding models and regulatory frameworks.

Data on healthcare infrastructure, funding models and other relevant factors were obtained from the OECD Health Statistics database.^11^ This database provides a comprehensive set of indicators on health and healthcare systems across multiple countries.

### Data analysis

The data obtained from the literature review, personal communication and OECD database were analysed using a thematic analysis approach. The data were organized into themes related to the delivery of OPAT across different countries, including organizational structures, funding models and regulatory frameworks. The data were then compared and synthesized to identify similarities and differences in OPAT delivery across different countries.

In order to systemically compare international variation in OPAT programmes we chose aspects of OPAT care that are considered benchmarks or best practice as previously proposed within the concept of the ‘OPAT Bundle’:^12^ patient selection; infection specialist involvement; patient/family education; care transition; outpatient monitoring; and OPAT programme measures.

## Variances in OPAT structure

### Centralized programmes

#### Ireland

The Irish national OPAT programme was established in 2013 and is a centralized system coordinating patients’ OPAT care.^10^ It accepts referrals from all public hospitals within the Republic of Ireland. The service is entirely funded by the Irish Health Service Executive (HSE) and provided free of charge to all users. Primary care doctors can make referrals, but this mechanism is not widely used. Prior to the establishment of the national programme, OPAT services were provided on an *ad hoc*, per-hospital basis without standardization of practice.^13^

Over half of the population of Ireland also avail themselves of private health insurance;^14^ there are three fully private providers who do not use the national centralized system but provide care in line with national guidelines.^15–17^

Hospital-administered OPAT (H-OPAT) is delivered by nurse-led community intervention teams, which are predominantly HSE teams, although in some geographical areas the service is provided by private providers contracted to the HSE. Self-administered OPAT (S-OPAT) is where the patients, or their carers, administer the antimicrobial(s). All patients are reviewed on a weekly basis by the prescribing team as per the Irish guidelines, in keeping with the OPAT bundle.^10,12^

Accurate national-level summary data incorporating all OPAT referrals is collated by the National Programme, something unique compared with other countries. These registry data are now 10 years old and provides a valuable insight into national prescribing patterns. Since the inception of the registry from 2013 to 2023, there have been 17 558 patient episodes over 42 health centres. This corresponds to over 292 860 OPAT treatment days. The Irish health system operates at a high percentage of occupied bed capacity, which may explain the relatively high use of OPAT comparative to Ireland’s population (5.1 million).^18^

The Irish OPAT system has fully operationalized the concept of the OPAT bundle. Patient-specific factors such as physical, social and logistic criteria form part of the initial assessment.^10^ Patients must be seen and assessed by an infection specialist both before and during OPAT. This forms part of the care transition pathway and also ensures that antimicrobial stewardship is a core component of the OPAT programme.^10^ Patient education is of paramount importance, particularly for S-OPAT, and is part of the OPAT assessment.^10^

Finally, using the national registry, outcome measurement is built into the programme, which acts as a valuable dataset to assess OPAT success, as well as to identify areas for clinical and service improvement.

## Decentralized programmes

###  

#### Brazil

The Brazilian healthcare system, Sistema Único de Saúde (SUS), is a tax-funded universal system providing free healthcare services to all citizens. Established in 1988, SUS is a decentralized system with federal, state and municipal levels of management. It covers primary, secondary and tertiary care.^19^ Additionally, a private healthcare sector coexists alongside SUS, offering services to those who can afford private insurance or direct payments.

Home care is also a specific offering within SUS, but at present it does not offer OPAT via this service.

OPAT appears to be a relatively new development in Brazil based on the published literature, with the first pilot programme initiated in 2013 but with rapid growth since. A recent national review paper notes that within the public system, ambulatory care units and day hospitals are the primary OPAT delivery mechanisms. Some private providers do operate OPAT within Hospital at Home systems, although on a limited basis.^20^

The Brazilian Infectious Diseases Society convened a group of national experts in 2017 to determine national recommendations for OPAT that were subsequently published by the society.^21^ These guidelines make similar recommendations to IDSA and incorporate all aspects of the OPAT bundle apart from outcome and registry monitoring.

There have been multiple papers showing the possible benefits of OPAT to the Brazilian healthcare system, including saving bed days and realizing benefit for antimicrobial stewardship.^22,23^

#### Canada

In Canada, healthcare is funded through a decentralized healthcare insurance model and is administered at a provincial level, resulting in a variety of care delivery approaches.^24^

All Canadian citizens and permanent residents receive healthcare coverage for medically necessary hospital and physician services, However approximately 66% of Canadians are also in possession of private healthcare insurance.^25^

There are currently very few published data on OPAT availability and use in Canada. There is no national programme and availability seems to be through major academic teaching hospitals. There are no reliable published data on overall national usage, and studies appear to be limited to descriptions of individual institutional or regional programmes.^26,27^ When patients are discharged on OPAT, the cost of other drugs and supplies is guaranteed for public patients.^28^ This is likely a significant barrier to the development of OPAT programmes.

For the individual Canadian hospitals that operate OPAT programmes, their published data show high levels of patient satisfaction and cost savings, suggesting there is scope for the wider expansion of OPAT services.^26,29^ However, the lack of a formal national programme and national guidelines make an assessment of OPAT use in Canada challenging.

#### Italy

Italy’s National Health Service automatically covers all citizens and legal foreign residents. It provides free primary and inpatient care. Co-payments are required for speciality visits and procedures, as well as some outpatient drugs.^30^ There are 19 separate regions and two autonomous provinces, which are responsible for delivering care through over 100 local health units.^31^

OPAT has long been established in Italy; however, it differs in some respects to other European countries. Firstly, while OPAT can be prescribed by a primary care doctor, there is a stipulation that only doctors can administer the antibiotic in the patient’s home. This is an obvious impediment to service expansion.^32^ Furthermore, the number of antibiotics that can be prescribed for community use is limited to a list authorized by the Italian Medicines Agency.^33^ A smaller list of reserved antimicrobials can be administered in the community under specialist supervision for the continuation of tertiary-level care.^33^

Italy was part of the now defunct International OPAT registry set up by IDSA. A limited number of sites participated in the registry and a number of publications arose demonstrating changing trends of antimicrobial prescribing.^34^

Within the scope of the literature search, there were no published national guidelines containing programmatic or quality measures. However, there are clear efforts at antimicrobial stewardship (AMS) via the controlled use of community antimicrobials.^35^

#### Singapore

Singapore’s healthcare system is characterized by a unique blend of public and private elements, emphasizing significant personal responsibility through mandatory health savings accounts called *Medisave*. The system is funded through a combination of government subsidies, individual savings and insurance. It provides universal coverage with a significant degree of government regulation to ensure accessibility and cost control.^36^

OPAT services were first introduced to Singapore in 2002, and since that time most of Singapore’s public hospitals have introduced programmes on a decentralized basis.

The common model is one where patients attend outpatient centres, although provision exists for homecare-based delivery for patients with mobility issues.^37^

Although OPAT in Singapore is decentralized, different academic hospitals have combined and published their data to give an overview of its OPAT utilization. Singapore maintains a combined outcomes registry, allowing for overall evaluation of the service across the involved provider sites. Singapore has fully implemented all aspects of the OPAT bundle.^38^

An interesting aspect of Singapore’s OPAT programmes is that the health system’s financing structure disincentivizes OPAT. These programmes attract no subsidies and impose limitations on Medisave usage. Despite these challenges, OPAT remains a cost-saving practice. However, the current financing structure likely limits the programme’s growth.^37^

#### UK

The UK’s National Health Service (NHS) is a publicly funded healthcare system providing free medical care to residents at the point of use. Established in 1948, it is funded through taxation and managed by the Department of Health and Social Care. The NHS is divided into four regional systems for England, Scotland, Wales and Northern Ireland, each responsible for their own healthcare services.

Despite the existence of the NHS, there is currently no centralized OPAT programme in the UK. There are more than 80 services, each offering OPAT, and until 2020 were voluntarily feeding information into a centralized registry maintained by BSAC.^39^ All services contributing to the database could compare their own data with other contributing services at a national level. Use of the registry was not compulsory, but it did deliver the most comprehensive picture of OPAT provision available nationally. The registry system closed in March 2020 and was replaced with a National Service Directory, which aims to provide a comprehensive overview of OPAT services and key OPAT personnel.

Good practice guidelines for the use of OPAT were published in 2012.^9^ These guidelines reflected the OPAT bundle but were tailored to the NHS.

A recent paper published by BSAC gives an excellent overview of the current scale of the OPAT programme. Registry data from 2015 to 2019 encompassing 27 481 patient episodes across 57 health centres corresponded to over 442 280 OPAT treatment days.^39^ Currently services are offered primarily through hospitals and on a much smaller scale within the primary healthcare network.^40^

The NHS in Scotland, where health matters are devolved, are currently developing their own national registry, which will collate all details of care and outcomes across the country.^41^

An economic assessment comparing OPAT versus inpatient care for six diagnoses extracted from the national registry data has shown that on average the cost of OPAT is 25%–50% less, demonstrating potential economic benefits.^42^

While the UK has incorporated many aspects of the OPAT bundle into their national recommendations, there is no centralized OPAT programme and therefore governance and oversight of these practices lies within individual hospitals and primary healthcare networks who provide OPAT care.^9^ As there is no current mandatory UK registry, collating national clinical outcome and programme measures remains challenging.

#### USA

Despite being one of the first countries to offer OPAT services, the current system in the USA is relatively fragmented owing to the complex healthcare funding streams.^43,44^ Healthcare provision is heavily reliant on insurance coverage, resulting in a mix of public and private providers, insurers and payers. Public programmes exist, including *Medicare* for seniors and *Medicaid* for low-income individuals.

The result is significant disparities in access to OPAT dependent on health insurance. For approximately 60 million patients dependent on the government-run national health insurance *Medicare*, reimbursement is only available for OPAT on days when a health provider attends the home.^45^

Notwithstanding that, OPAT is widely used and advertised as a patient-serving benefit. In 1997, IDSA published their first set of OPAT guidelines and introduced an OPAT registry, which was discontinued 3 years later due to a lack of funding. At the time of its operation, it contained information from 24 contributing sites in the USA, detailing the care of more than 8000 patients who received greater than 11 000 antibiotic courses between 1997 and 2000. There is now no accurate up-to-date data on OPAT usage although previous work suggested that annually approximately 250 000 Americans receive OPAT.^46^

Given the lack of systemically collected data in the USA, it is difficult to assess national measures of compliance with a structure like the conceptual OPAT bundle. Certainly, the IDSA guidelines incorporate all aspects of the bundle and there are multiple publications involving individual hospital OPAT programmes that demonstrate best practice within their own institutions.^47,48^ The loss of the national registry makes outcome and programmatic assessment difficult, and its re-introduction would be welcomed.^49^

### Hospital at Home

#### Australia

Australia’s healthcare system blends public and private services, providing residents with universal health coverage through a national system called *Medicare*. Established in 1984, *Medicare* offers free or subsidized treatment by healthcare providers and free care in public hospitals. A substantial number of Australians also opt for private health insurance.

OPAT was established in Australia in 1994 following a pilot programme, Hospital in the Home (HITH); it has since become established as a standard of care.^50^ OPAT services are funded by *Medicare* with no additional cost to a patient.

Demand for OPAT is growing, and is being encouraged by the Australian Department of Health.^51^ Between 2011 and 2017, OPAT patient ‘admissions’ grew by almost twice the rate of all general hospital admissions in a group of 20 principle referring hospitals.^52^ For the 1 year period 2017–18, greater than 595 000 OPAT care days were delivered for public patients in Australia, accounting for over 5% of acute-care bed days.^53^

Australia conducted an independent review of HITH services in 2009, which concluded that as a well-established model of care, HITH is safe, effective and highly valued by patients, carers and staff.^54^

There are no published national OPAT guidelines, however, individual states operating HITH programmes have considered some aspects of the OPAT bundle.^54^ Guidelines for patient selection, education and care transition are clear; however, there is no mandate to have infection specialist involvement. Additionally, although there is a national HITH registry, the scope of these schemes goes beyond OPAT, resulting in fewer programmatic assessments, and no easily accessible data pertaining to OPAT specific outcomes.

#### France

The French healthcare system is a social health insurance system.^55^ It provides comprehensive coverage to all residents through mandatory health insurance, financed by employer and employee payroll taxes.

Citizens have the freedom to choose providers and are reimbursed for a significant proportion of their medical expenses. Optional private insurance is available to cover additional costs. OPAT is included in the coverage provided by the statutory health insurance system.

France began to offer OPAT services via hospital-at-home services, L’Hospitalisation á Domicile, in 1957.^56^ It should be noted that French Hospital at Home services are broad-ranging and a centralized service like that of the Irish system. While OPAT is a significant service, it is fourth in terms of bed days saved after complex dressing, enteral nutrition and complex nursing care for patients with obesity.^57^ While the service is widely available there is regional disparity of accessibility.^58^

A relatively unique feature of the French OPAT system is that GPs regularly utilize OPAT.^59^ The most up-to-date published figures for 2017 show that almost 300 000 bed days were saved through OPAT, with a year-on-year growth of approximately 6%.^60^

There have been limited studies of the cost effectiveness of OPAT within the French system but the majority of the relatively small studies to date demonstrate significant cost savings in the range of €15 000 per infective endocarditis patient and almost €45 000 per patient for the treatment of osteomyelitis.^61,62^

Considering the French system through the lens of the OPAT bundle, it performs well. There is a national federation of Hospital at Home services, which conducts monitoring and issues guidance for Hospital at Home activities, including OPAT patient selection and education. However, there is no requirement for an infection specialist to be involved nor is there national guidance related to outpatient monitoring. Finally, while there is a national Hospital at Home registry, the data published solely pertain to admission avoidance. There is no published literature on national outcomes or quality indicators pertaining solely to OPAT.^57^

#### Spain

Spain’s healthcare system is a universal public system known as the Spanish National Health System [Sistema Nacional de Salud (SNS)]. It provides comprehensive healthcare services to all residents free at the point of care, funded primarily through taxation. The system is decentralized, giving regional governments significant control over their healthcare services^63^ Within this service, Spain’s Home Hospitalization Unit, founded in 1981, provides OPAT in addition to other specialized hospital level care at home. The service exists across Spain delivered by over 100 home hospitalization units. OPAT is well established in Spain and there are good national-level data for both its efficacy and cost-saving potential.^64^ Referrals are accepted from both primary and secondary care. There is also an active Society for Home Hospitalization, which organizes and presents OPAT research. They have produced evidence and expert-based guidelines with a view to standardizing OPAT care within Spanish Hospital at Home settings.^65^

Spain has an active OPAT registry that began in 2011. It was conceived as a multicentre project but does not appear to contain information from every participating Home Hospitalization Unit.^66^ A significant study published in 2017 involving 1190 patients who received OPAT via a Home Hospitalization Unit demonstrated reduced costs of up to 81%.^67^

Spain has widely adopted the concept of the OPAT bundle and consideration is given to all aspects of patient selection, education and care transition. Infectious diseases is not a formal speciality *per se* in Spain, but the society makes active reference to microbiological specialist involvement. The national registry also publishes outcome data but outcomes are not limited to OPAT, which is just a component of the Hospital at Home strategy.^68^

### Pilot OPAT programmes

#### Germany

The German healthcare system works on a mandatory insurance model, where insurance, either statutory or private, is mandated for all citizens and permanent residents. The statutory model mandates inpatient, outpatient and prescription drug coverage to German residents.^69^

Due to a lack of statutory authorization, OPAT within Germany can be subject to unfavourable reimbursement arrangements, which is widely seen as a barrier to its wider adoption.^70^ Compared with France, its nearest comparator nation, OPAT is much less widely used. When it is used it tends to be for specified clinical conditions such as cystic fibrosis.

A pilot clinical trial in Cologne, Germany, commencing in October 2019 but recently published, was set up to ascertain the feasibility of OPAT provision on the basis that it is used very infrequently despite its benefits.^70^ Authors postulated this is due to an inadequate knowledge of OPAT as a model of care, as well as deficits in the outpatient care structure where OPAT is not reflected in the remuneration system. The study concluded that OPAT can be safely and effectively provided and advised the creation of formal guidelines, financial and structural regulations, and enhancement of multidisciplinary team engagement to enable the expansion of OPAT provision.^71^

## Discussion

Despite the documented efficacy and cost-effectiveness of OPAT, there is wide intercountry variation in its use and uptake.

### Healthcare system structures

The structure of the overall health system likely plays a role; there is a probable drive to save bed days in countries such as Ireland and the UK, which have a comparatively low number of beds per 100 000 population.^18,72^ By contrast, in countries such as Germany, which have a low rate of bed occupancy use by international comparison, there is less likely to be intrinsic demand to create bed availability.

Based on the literature, integration of OPAT within Hospital at Home-type services, seems to also allow for the wider and broader adoption of OPAT services in general.

Furthermore, the use of centralized OPAT programmes, where they can be implemented within a health system, allows for further systematic evaluation of services, and easier implementation of audit and quality improvement systems.

### Variability in cost savings

The demand for cost savings may also contribute to OPAT demand. In health systems that are publicly funded there is almost always a greater demand for services than available supply. If a system can safely and effectively treat a patient in their own home, there is a strong financial incentive to generate savings that can be used to finance other aspects of care, as well as the moral perspective to maximize the use of public resources.

The cost savings in OPAT are largely generated through avoiding inpatient stays. The cost of OPAT is variable depending on the mode of administration chosen but can be minimal if options such as patient self-administration of antimicrobials is explored. There have been cost-effectiveness analyses published in most of the countries outlined above, which all demonstrate OPAT as a definitive cost-saving modality.^5,42,61,62^ Cost savings are likely to be greater in countries where inpatient care is relatively more expensive such as the USA.^6^

A further significant financial and clinical benefit of OPAT is the reduction in risk of healthcare-acquired infection.^73^ By removing a patient with an indwelling vascular access device from a ward environment, their risk of acquiring an MDRO significantly reduces.^74^ MDROs are a significant cause of morbidity and substantially increase healthcare costs.

### Quantitative assessment

The lack of adequate national registers makes intercountry comparisons of OPAT difficult. There are survey data showing that OPAT use is minimal in certain countries but the reasons behind this have not been directly explored.^38^

However, in countries such as Ireland, and to a lesser extent France, where there are published usage data, we do know that there is intracountry variation. In Ireland and France, there are geographical variations in OPAT use, which need to be addressed to provide equity of access across the population.^58^

There is a lack of peer-reviewed data specifically related to the equity of access to OPAT care, but it is reasonable to assume that prescriber knowledge, comfort and experience of OPAT are likely drivers of use.

### Clinical efficacy

When it comes to the clinical efficacy of OPAT, there are limited studies looking at performance within individual centres and direct country-level comparisons. The only published direct country comparison comes from work derived from the now defunct international OPAT registry commenced by IDSA. The ability to draw international comparisons are limited by the dominance of the 24 US sites compared with 8 and 3 in Italy and the UK, respectively. There was no significant difference in clinical outcome in this limited comparison but a large difference in the average duration of OPAT therapy, with a 56 day average in Italy, 22.5 days in the USA and 19.9 days in the UK.^75^

### AMS and OPAT

AMS is an increasingly important aspect of modern healthcare. With the expansion of OPAT, there is a need to incorporate good AMS practice into patient care. While ideally an antibiotic should be prescribed in terms of direct susceptibility and most directed spectrum, we do know that a large amount of prescribing is done in terms of empirical coverage.^76^

Looking across countries, many do not mandate infection specialist involvement; this is a clear lost opportunity for AMS. However, there are examples of good AMS practice internationally that we identified, such as the requirement in Ireland to include infection specialists in all OPAT decisions, and additional approvals required for OPAT exceeding specific durations or the prescription of antifungal therapy.

There is also a clear need to consider variations in prescription duration in clinical practice internationally. As evidence mounts that shorter durations of antimicrobial therapy are efficacious, there is a need to ensure OPAT practitioners alter practice in keeping with this evidence.^77,78^

There is also evidence that logistical factors contribute to the provision of OPAT care.^79^ Analysis of the Irish national registry data suggests that required frequency of administration likely does play a role in antimicrobial selection.^80^ There is a need to develop infusion delivery systems so that the most appropriate antimicrobial choices can also be offered to OPAT patients, rather than making a choice based on delivery frequency.

Moreover, the most common route of administration of OPAT varies from country to country. For example, Italy has a very high use of injectable medications for outpatient management. Over 53% of outpatient lower respiratory tract infections are treated with parenteral antimicrobial treatment, compared with a rate of 0.2% in the UK. Italy also makes greater use of intramuscular injections.^81^ This may be because parenteral treatment is considered by the population to be a more effective treatment than an oral option. These cultural and/or patient preferences have cost-effectiveness implications, as well as considerations for the safe, effective and cost-efficient delivery of OPAT.^82,83^

### Complex outpatient antimicrobial therapy (COpAT)

Over recent years, COpAT has become a rapidly developing area of practice. While the term COpAT does not specifically denote oral or parenteral treatment, the literature in the area tends to associate it with ‘oral only’ treatment.^84^ However, this definition neglects the fact that traditional OPAT care itself is becoming more complex. Data from the Irish registry shows that COpAT, (defined as ‘the use of more than two antimicrobials and/or OPAT exceeding 6 weeks in duration and/or the use of antifungal or antiviral medications) has grown year on year, peaking at over 310 cases in 2022 (unpublished data).

While there is increasing evidence that shows equivalence with oral and parenteral treatment for certain infections,^85,86^ in the era of MDROs, there will remain a cohort of patients for whom IV therapy is the only available treatment.

Notwithstanding this, there is an undeniable trend and benefit towards moving towards oral regimens as efficacy evidence accumulates. Accompanying this is uncertainty around how complex antimicrobial regimens can be safely and effectively managed in the outpatient setting.

Given that OPAT has been proved to be a safe care model across multiple jurisdictions, there are opportunities to use the systems and safety measures in place for OPAT to provide follow-up of patients on complex regimens.^87^ The safety structures that have emerged through OPAT delivery mean that patients being treated with complex oral regimens could easily be facilitated with current OPAT structures, whilst ensuring that patient education, infection specialist involvement and regular follow-up and monitoring become an immediate and standard part of COpAT care. This is particularly important for drugs within the oxazolidinone and quinolone classes, often used within COpAT and which offer oral antimicrobial coverage for common resistant organisms. Non-infection specialists may not be aware of the potential toxicities and complications associated with prolonged antimicrobial usage, and it is therefore highly probable that for this cohort of patients they would be best served by integrating COpAT within existing OPAT services.

## Limitations

In many jurisdictions, the lack of use or existence of an OPAT registry required to effectively compare within and between regions and nations is a significant challenge to comparative review. While multiple countries chosen in this analysis are not comparable in terms of population size or density, or geographical distribution of population, many similar countries are difficult to compare due to a lack of publications or availability of national-level published data. There is a lack of publications from countries in South and Central America, Africa, Oceania and Asia, which shows there is a need for an accurate up-to-date assessment of the current and actual use of OPAT internationally to gauge the scope for wider adoption and quality improvement.

Because of our search strategy, papers relating to outpatient antimicrobial therapy outside of the home and outside of the outpatient department, e.g. primary care centres, pharmacy, may have been missed.

It is possible that the real-life implementation of OPAT in many regions and the documented implementation of OPAT in the literature varies significantly. Furthermore, in terms of the healthcare providers who contributed to the paper, it is possible that there was a source of bias, for example those who contributed were experts in OPAT provision.

### Conclusions

This paper has highlighted significant international variations in OPAT structures, delivery options and usage, emphasizing the need for further examination, standardization and improvement of these programmes. Factors contributing to these variations include healthcare system structures, cost-savings demands and regional differences. There is also a clear need to integrate AMS within any OPAT or Hospital at Home strategy. Comprehensive registries and well-designed clinical trials are needed to facilitate intercountry comparisons and determine optimal treatment durations for multiple infections.

COpAT will most likely continue to increase in the future, and integration of this delivery mechanism should be considered in all countries that operate an OPAT programme.

By identifying areas of variation and exploring opportunities for improvement, we can enhance patient outcomes, reduce healthcare costs, and optimize resource utilization. Future research should focus on creating adaptable and scalable OPAT models that can be applicable across different healthcare systems, such as the OPAT care bundle, thereby maximizing the potential benefits of outpatient antimicrobial therapy for patients, hospitals and wider society.

## References

[1] Williams DN, Baker CA, Kind AC et al The history and evolution of outpatient parenteral antibiotic therapy (OPAT). Int J Antimicrob Agents 2015; 46: 307–12. 10.1016/j.ijantimicag.2015.07.001 26233483

[2] Berrevoets MAH, Oerlemans AJM, Tromp M et al Quality of outpatient parenteral antimicrobial therapy (OPAT) care from the patient’s perspective: a qualitative study. BMJ Open 2018; 8: e024564. 10.1136/bmjopen-2018-024564 PMC6252647 30420352

[3] Wee LE, Sundarajoo M, Quah WF et al Health-related quality of life and its association with outcomes of outpatient parenteral antibiotic therapy. Eur J Clin Microbiol Infect Dis 2020; 39: 765–72. 10.1007/s10096-019-03787-6 31873862

[4] Bugeja SJ, Stewart D, Vosper H. Clinical benefits and costs of an outpatient parenteral antimicrobial therapy service. Res Social Adm Pharm 2021; 17: 1758–63. 10.1016/j.sapharm.2021.01.009 33551209

[5] Kwok CS, Whittaker JJ, Malbon C et al Outpatient parenteral antimicrobial therapy (OPAT) service is associated with inpatient-bed cost savings. Br J Cardiol 2021; 28: 38. 10.5837/bjc.2021.038 35747699 PMC8988795

[6] Psaltikidis EM, da Silva EN, Bustorff-Silva JM et al Economic evaluation of outpatient parenteral antimicrobial therapy: a systematic review. Expert Rev Pharmacoecon Outcomes Res 2017; 17: 355–75. 10.1080/14737167.2017.1360767 28776441

[7] Korenstein D, Husain S, Gennarelli RL et al Impact of clinical specialty on attitudes regarding overuse of inpatient laboratory testing. J Hosp Med 2018; 13: 844–7. 10.12788/jhm.2978 29964278 PMC6265055

[8] Norris AH, Shrestha NK, Allison GM et al 2018 Infectious Diseases Society of America clinical practice guideline for the management of outpatient parenteral antimicrobial therapy. Clin Infect Dis 2019; 68: e1–35. 10.1093/cid/ciy867 30423035

[9] Chapman ALN, Patel S, Horner C et al Updated good practice recommendations for outpatient parenteral antimicrobial therapy (OPAT) in adults and children in the UK. JAC Antimicrob Resist 2019; 1: dlz026. 10.1093/jacamr/dlz026 34222901 PMC8209972

[10] Sweeney E, Curtin N, de Barra E et al National guidelines on the provision of outpatient parenteral antimicrobial therapy (OPAT). Ir Med J 2020; 113: 123. https://imj.ie/national-guidelines-on-the-provision-of-outpatient-parenteral-antimicrobial-therapy-opat/35575598

[11] OECD . OECD Health Statistics 2022. https://www.oecd.org/els/health-systems/health-data.htm.

[12] Muldoon EG, Snydman DR, Penland EC et al Are we ready for an outpatient parenteral antimicrobial therapy bundle? A critical appraisal of the evidence. Clin Infect Dis 2013; 57: 419–24. 10.1093/cid/cit211 23572486 PMC3703103

[13] Muldoon EG, Allison GM, Gallagher D et al Outpatient parenteral antimicrobial therapy (OPAT) in the republic of Ireland: results of a national survey. Eur J Clin Microbiol Infect Dis 2013; 32: 1465–70. 10.1007/s10096-013-1899-4 23728737 PMC3973129

[14] The Health Insurance Authority . The Irish Healthcare System: An Historical and Comparative Review. 2018; 94. https://www.hia.ie/sites/default/files/The%20Irish%20Healthcare%20System%20-%20An%20Historical%20and%20Comparative%20Review.pdf.

[15] VHI . Hospital at Home. https://www1.vhi.ie/members/hospital-at-home.

[16] Laya Healthcare . Yourcare@home. https://www.layahealthcare.ie/yourcareathome/.

[17] Irish Life health . Health in the Home. https://www.irishlifehealth.ie/benefits/key-benefits/health-in-the-home.

[18] Keegan C, Brick A, Walsh B et al How many beds? Capacity implications of hospital care demand projections in the Irish hospital system, 2015-2030. Int J Health Plann Manage 2019; 34: e569–82. 10.1002/hpm.2673 30277279

[19] Commonwealth Fund . International Health Care System Profiles—Brazil. 2020. https://www.commonwealthfund.org/international-health-policy-center/countries/brazil.

[20] Oliveira PR, Carvalho VC, Uip DE et al Outpatient parenteral antimicrobial therapy in Brazil. Ther Adv Infect 2023; 10: 20499361231178625. 10.1177/20499361231178625 PMC10251471 37304574

[21] Oliveira PR, Carvalho VC, Cimerman S et al Recommendations for outpatient parenteral antimicrobial therapy in Brazil. Braz J Infect Dis 2017; 21: 648–55. 10.1016/j.bjid.2017.06.006 28711455 PMC9425540

[22] Oliveira PR, da Silva Felix C, de Carvalho VC et al Outpatient parenteral antimicrobial therapy for orthopedic infections—a successful public healthcare experience in Brazil. Braz J Infect Dis 2016; 20: 272–5. 10.1016/j.bjid.2016.03.005 27102779 PMC9425536

[23] Cassettari V, Novato N, Onuchic MHF. Antimicrobial stewardship in the outpatient parenteral antimicrobial therapy (OPAT) setting: the impact of prescription assessment by an infectious diseases specialist. Braz J Infect Dis 2021; 25: 101560. 10.1016/j.bjid.2021.101560 33716018 PMC9392152

[24] European Observatory on Health Systems and Policies . Canada: health system review 2020. 2020. https://eurohealthobservatory.who.int/publications/i/canada-health-system-review-2020.

[25] Martin D, Miller AP, Quesnel-Vallée A et al Canada’s universal health-care system: achieving its potential. Lancet 2018; 391: 1718–35. 10.1016/S0140-6736(18)30181-8 29483027 PMC7138369

[26] Staples JA, Ho M, Ferris D et al Outpatient versus inpatient intravenous antimicrobial therapy: a population-based observational cohort study of adverse events and costs. Clin Infect Dis 2022; 75: 1921–9. 10.1093/cid/ciac298 35439822

[27] Azhir A, Chapman M. Delivery models, efficacy, safety, and cost reduction of outpatient parenteral antimicrobial therapy in British Columbia. BCMJ 2022; 64: 160–5. https://bcmj.org/articles/delivery-models-efficacy-safety-and-cost-reduction-outpatient-parenteral-antimicrobial

[28] The Commonwealth Fund . International Health Care Systems Profiles: Canada. 2020. https://www.commonwealthfund.org/international-health-policy-center/countries/canada.

[29] Yadav K, Kumar S, Chhabra S et al Outpatient parenteral antibiotic therapy (OPAT) and inpatient treatment strategies for emergency department patients with cellulitis: a cost analysis. CJEM 2022; 24: 520–8. 10.1007/s43678-022-00320-1 35675027

[30] European Observatory on Health Systems and Policies . Italy health system information. https://eurohealthobservatory.who.int/countries/italy.

[31] Cicchetti A, Gasbarrini A. The healthcare service in Italy: regional variability. Eur Rev Med Pharmacol Sci 2016; 20(Suppl 1): 1–3. https://www.europeanreview.org/article/11987 28083867

[32] Esposito S . [Outpatient parenteral antibiotic treatment: the Italian model]. Infez Med 2001; 9: 7–12. 12082343

[33] Italian Medicine Agency . Manuale antibiotici AWaRe. Edizione italiana del ‘The WHO AWaRe Antibiotic Book’. 2023. https://www.aifa.gov.it/documents/20142/1811463/Manuale_antibiotici_AWaRe.pdf.

[34] Esposito S, Ianniello F, Noviello S et al [Outpatient Parenteral Antibiotic Therapy (OPAT): the Italian registry]. Infez Med 2002; 10: 169–75. 12704268

[35] Esposito S . Treatment of lower respiratory tract infections in Italy: the role of outpatient parenteral antibiotic therapy. Chemotherapy 2001; 47 Suppl 1: 33–40. 10.1159/000048566 11096187

[36] Tan CC, Lam CSP, Matchar DB et al Singapore’s health-care system: key features, challenges, and shifts. Lancet 2021; 398: 1091–104. 10.1016/S0140-6736(21)00252-X 34481560

[37] Fisher DA, Kurup A, Lye D et al Outpatient parenteral antibiotic therapy in Singapore. Int J Antimicrob Agents 2006; 28: 545–50. 10.1016/j.ijantimicag.2006.08.018 17097856

[38] Fisher D, Michaels J, Hase R et al Outpatient parenteral antibiotic therapy (OPAT) in Asia: missing an opportunity. J Antimicrob Chemother 2017; 72: 1221–6. 10.1093/jac/dkw551 28077673

[39] Gilchrist M, Barr D, Drummond F et al Outpatient parenteral antimicrobial therapy (OPAT) in the UK: findings from the BSAC National Outcomes Registry (2015–19). J Antimicrob Chemother 2022; 77: 1481–90. 10.1093/jac/dkac047 35187565

[40] Chapman ALN . Outpatient parenteral antimicrobial therapy. BMJ 2013; 346: f1585. 10.1136/bmj.f1585 23532865

[41] Scottish Antimicrobial Prescribing Group . Outpatient parenteral antimicrobial therapy (OPAT). https://www.sapg.scot/guidance-qi-tools/outpatient-parenteral-antimicrobial-therapy-opat/.

[42] Dimitrova M, Gilchrist M, Seaton RA. Outpatient parenteral antimicrobial therapy (OPAT) versus inpatient care in the UK: a health economic assessment for six key diagnoses. BMJ Open 2021; 11: e049733. 10.1136/bmjopen-2021-049733 PMC8479950 34588251

[43] Rucker RW, Harrison GM. Outpatient intravenous medications in the management of cystic fibrosis. Pediatrics 1974; 54: 358–60. 10.1542/peds.54.3.358 4213282

[44] Nolet BR . Update and overview of outpatient parenteral antimicrobial therapy regulations and reimbursement. Clin Infect Dis 2010; 51: S216–9. 10.1086/653522 20731579

[45] Streifel AC, Sikka MK. The urgent need for Medicare reimbursement for home infusion antibiotics amidst a pandemic. Clin Infect Dis 2020; 71: 3250–1. 10.1093/cid/ciaa658 32463077 PMC7314214

[46] Tice AD, Rehm SJ, Dalovisio JR et al Practice guidelines for outpatient parenteral antimicrobial therapy. Clin Infect Dis 2004; 38: 1651–71. 10.1086/420939 15227610

[47] Agnihotri G, Gross AE, Seok M et al Decreased hospital readmissions after programmatic strengthening of an outpatient parenteral antimicrobial therapy (OPAT) program. Antimicrob Steward Healthc Epidemiol 2023; 3: e33. 10.1017/ash.2022.330 36865701 PMC9972539

[48] Madaline T, Nori P, Mowrey W et al Bundle in the Bronx: impact of a transition-of-care outpatient parenteral antibiotic therapy bundle on all-cause 30-day hospital readmissions. Open Forum Infect Dis 2017; 4: ofx097. 10.1093/ofid/ofx097 28852672 PMC5570156

[49] Muldoon EG, Switkowski K, Tice A et al A national survey of infectious disease practitioners on their use of outpatient parenteral antimicrobial therapy (OPAT). Infect Dis 2015; 47: 39–45. 10.3109/00365548.2014.967290 25415655

[50] Ruth D, Greenberg PB, Campbell DA. Evaluating health-care delivery: Hospital in the Home. Intern Med J 2001; 31: 135–7. 10.1046/j.1445-5994.2001.00046.x 11478340

[51] Li W, Branley J, Sud A. Outpatient parenteral antibiotic therapy in a suburban tertiary referral centre in Australia over 10 years. Infection 2018; 46: 349–55. 10.1007/s15010-018-1126-4 29464675

[52] Montalto M, McElduff P, Hardy K. Home ward bound: features of hospital in the home use by major Australian hospitals, 2011–2017. Med J Aust 2020; 213: 22–7. 10.5694/mja2.50599 32356602

[53] Swannell C . MJA News. Hospital in the Home: a growing part of Australian care. 2020. https://www.mja.com.au/journal/2020/hospital-home-growing-part-australian-care.

[54] Victoria Department of Health . Hospital in the Home. https://www.health.vic.gov.au/patient-care/hospital-in-the-home.

[55] Chevreul K, Berg Brigham K, Durand-Zaleski I et al France: health system review. Health Syst Transit 2015; 17: xvii. https://eurohealthobservatory.who.int/publications/i/france-health-system-review-2015. 26766545

[56] de Stampa M, Andrieu M, Chataux S et al Hospitalisation à domicile et malades âgés [Hospital at home and elderly patients]. NPG Neurol Psychiatr Gériatr 2014; 14: 265–9. 10.1016/j.npg.2014.02.012

[57] DGOS, Ministère de la Santé et de la Prévention . L’hospitalisation à domicile. 2023. https://sante.gouv.fr/soins-et-maladies/prises-en-charge-specialisees/had-10951/had.

[58] Triffault-Fillit C, Ferry T, Perpoint T et al Outpatient parenteral antibiotic therapy: evaluation of practices and limits of use in rural areas in France. Med Mal Infect 2018; 48: 130–5. 10.1016/j.medmal.2017.09.008 29050864

[59] FNEHAD . Médecins généralistes. https://www.fnehad.fr/en2016/medecin-generaliste/.

[60] ATIH . Contenu des bases HAD 2017. https://www.atih.sante.fr/contenu-des-bases-had-2017.

[61] Bernard L, El-Hajj, Pron B et al Outpatient parenteral antimicrobial therapy (OPAT) for the treatment of osteomyelitis: evaluation of efficacy, tolerance and cost. J Clin Pharm Ther 2001; 26: 445–51. 10.1046/j.1365-2710.2001.00380.x 11722682

[62] Lacroix A, Revest M, Patrat-Delon S et al Outpatient parenteral antimicrobial therapy for infective endocarditis: a cost-effective strategy. Med Mal Infect 2014; 44: 327–30. 10.1016/j.medmal.2014.05.001 25022891

[63] European Observatory on Health Systems and Policies . Spain health system information. https://eurohealthobservatory.who.int/countries/spain.

[64] Hospital General Universitario Gregorio Marañón . Hospitalización a Domicilio. 2018. https://www.comunidad.madrid/hospital/gregoriomaranon/profesionales/servicios-medicos/hospitalizacion-domicilio.

[65] SEHAD (Spanish Society of Home Hospitalization) . Home parenteral antibiotic therapy. https://www.sehad.org/index.php/grupos-de-trabajo/antibioterapia-parenteral-domiciliaria.

[66] Mirón-Rubio M, González-Ramallo V, Estrada-Cuxart O et al Intravenous antimicrobial therapy in the hospital-at-home setting: data from the Spanish Outpatient Parenteral Antimicrobial Therapy Registry. Future Microbiol 2016; 11: 375–90. 10.2217/fmb.15.141 26974259

[67] González-Ramallo VJ, Mirón-Rubio M, Mujal A et al Costs of outpatient parenteral antimicrobial therapy (OPAT) administered by hospital at home units in Spain. Int J Antimicrob Agents 2017; 50: 114–8. 10.1016/j.ijantimicag.2017.02.017 28499957

[68] López Cortés LE, Mujal Martínez A, Fernández Martínez de Mandojana M et al Executive summary of outpatient parenteral antimicrobial therapy: Guidelines of the Spanish Society of Clinical Microbiology and Infectious Diseases and the Spanish Domiciliary Hospitalisation Society. Enferm Infecc Microbiol Clin 2019; 37: 405–9. 10.1016/j.eimc.2018.03.012 29784453

[69] European Observatory on Health Systems and Policies . Germany: health system review 2020. 2021. https://eurohealthobservatory.who.int/publications/i/germany-health-system-review-2020.

[70] ClinicalTrials.gov. Outpatient Parenteral Antibiotic Therapy in the Metropolitan Area of Cologne: NCT04002453. 2021. https://clinicaltrials.gov/ct2/show/NCT04002453.

[71] Schmidt-Hellerau K, Baade N, Günther M et al Outpatient parenteral antimicrobial therapy (OPAT) in Germany: insights and clinical outcomes from the K-APAT cohort study. Infection 2024; 10.1007/s15010-024-02199-9 PMC11289149 38478255

[72] OECD . Health at a Glance: Europe 2022: State of Health in the EU Cycle. 2022. https://www.oecd-ilibrary.org/social-issues-migration-health/health-at-a-glance-europe-2022_507433b0-en.

[73] Ng N, Bailey P, Pryor R et al Experiences in outpatient parenteral antimicrobial therapy (OPAT): barriers and challenges from the front lines. Antimicrob Steward Healthc Epidemiol 2021; 1: e42. 10.1017/ash.2021.213 36168502 PMC9495526

[74] Hauck K, Zhao X. How dangerous is a day in hospital? A model of adverse events and length of stay for medical inpatients. Med Care; 49: 1068–75. 10.1097/MLR.0b013e31822efb09 21945976

[75] Nathwani D . Ambulatory antimicrobial use: the value of an outcomes registry. J Antimicrob Chemother 2002; 49: 149–54. 10.1093/jac/49.1.149 11751779

[76] Gilchrist M, Seaton RA. Outpatient parenteral antimicrobial therapy and antimicrobial stewardship: challenges and checklists. J Antimicrob Chemother 2015; 70: 965–70. 10.1093/jac/dku517 25538169

[77] Spellberg B . The new antibiotic mantra—“Shorter Is Better”. JAMA Intern Med 2016; 176: 1254–5. 10.1001/jamainternmed.2016.3646 27455385 PMC5233409

[78] Spellberg B, Rice LB. Duration of antibiotic therapy: shorter is better. Ann Intern Med 2019; 171: 210–1. 10.7326/M19-1509 31284302 PMC6736742

[79] Steffens E, Quintens C, Derdelinckx I et al Outpatient parenteral antimicrobial therapy and antibiotic stewardship: opponents or teammates? Infection 2019; 47: 169–81. 10.1007/s15010-018-1250-1 30443780

[80] Reidy P, McWalter M, Muldoon E. Descriptive analysis of the Irish national outpatient parenteral antimicrobial therapy (OPAT) programme. Thirty-Second European Congress of Clinical Microbiology and Infectious Diseases (ECCMID), Lisbon, Portugal, 2022. https://2022.eccmid.org/abstracts/posters/. Poster 5001/L0222.

[81] Esposito S . Outpatient parenteral treatment of bacterial infections: the Italian model as an international trend? J Antimicrob Chemother 2000; 45: 724–7. 10.1093/jac/45.6.724 10837423

[82] Avorn J, Solomon DH. Cultural and economic factors that (mis)shape antibiotic use: the nonpharmacologic basis of therapeutics. Ann Intern Med 2000; 133: 128–35. 10.7326/0003-4819-133-2-200007180-00012 10896639

[83] Gaygısız Ü, Lajunen T, Gaygısız E. Socio-economic factors, cultural values, national personality and antibiotics use: a cross-cultural study among European countries. J Infect Public Health 2017; 10: 755–60. 10.1016/j.jiph.2016.11.011 28209467

[84] Seaton RA, Ritchie ND, Robb F et al From ‘OPAT’ to ‘COpAT’: implications of the OVIVA study for ambulatory management of bone and joint infection. J Antimicrob Chemother 2019; 74: 2119–21. 10.1093/jac/dkz122 30989175

[85] Li HK, Rombach I, Zambellas R et al Oral versus intravenous antibiotics for bone and joint infection. N Engl J Med 2019; 380: 425–36. 10.1056/NEJMoa1710926 30699315 PMC6522347

[86] Atkinson M, Lakhanpaul M, Smyth A et al Comparison of oral amoxicillin and intravenous benzyl penicillin for community acquired pneumonia in children (PIVOT trial): a multicentre pragmatic randomised controlled equivalence trial. Thorax 2007; 62: 1102–6. 10.1136/thx.2006.074906 17567657 PMC2094276

[87] Pertzborn M, Rivera CG, Tai DBG. Taking the route less traveled: on the way to COpAT. Ther Adv Infect Dis 2023; 10: 20499361231192771. 10.1177/20499361231192771 PMC10433885 37600977

